# Antiulcer activity of cod liver oil in rats

**DOI:** 10.4103/0253-7613.44152

**Published:** 2008-10

**Authors:** Salaj Khare, Mohammed Asad, Sunil S. Dhamanigi, V. Satya Prasad

**Affiliations:** Krupanidhi College of Pharmacy, Bangalore, India; 1 MNR Medical College, Andhra Pradesh, India

**Keywords:** Cod liver oil, duodenal ulcer, gastric cytoprotection, gastric secretion, gastric ulcer

## Abstract

**Objective:**

Cod liver oil is used widely as a dietary supplement. The present study was carried out to evaluate the effect of cod liver oil (0.5 g/kg, p.o. and 1 g/kg, p.o.) on gastric and duodenal ulcers.

**Materials and Methods:**

The study was carried out on different gastric ulcer models such as acetic acid induced chronic gastric ulcers, pylorus ligation, indomethacin induced ulcers, stress induced ulcers and ethanol induced ulcers. The duodenal ulcers were induced using cysteamine hydrochloride (HCl). Ranitidine (50 mg/kg p.o.) and misoprostol (100 *µ*g/kg, p.o.) were used as standard drugs.

**Results:**

Both doses of cod liver oil showed gastric ulcer healing effect in acetic acid induced chronic gastric ulcers, produced gastric antisecretory effect in pylorus-ligated rats and also showed gastric cytoprotective effect in ethanol-induced and indomethacin-induced ulcer. Cod liver oil also produced a significant reduction in the development of stress induced gastric ulcers and cysteamine induced duodenal ulcer. The high dose of cod liver oil (1 g/kg, p.o.) was more effective compared to the low dose (0.5 g/kg, p.o.).

**Conclusion:**

Cod liver oil increases healing of gastric ulcers and prevents the development of experimentally induced gastric and duodenal ulcers in rats.

## Introduction

Cod liver oil (CLO) is a dietary supplement that consists of polyunsaturated fatty acids (PUFA), which includes C_20_ fatty acids (17%), palmitoleic acid (7%) and C_22_ fatty acids (11%), vitamin A not less than 850 USP units/g and vitamin D not less than 85 USP units/g.[1,2] Cod liver oil is widely used in the treatment of rickets and osteomalacia as a dietary supplement.

The development and progression of gastric ulcer, to some extent, depends on the type of the food consumed by the patient. Studies have been carried out to specifically evaluate the effect of fish oils on the development of gastric ulcer. It has been reported that a diet rich in fish oils reduces arachidonic acid levels in the gastric mucosa and increases stress induced gastric erosions.[3] On the contrary, it is reported that fish oils rich in polyunsaturated fatty acids (PUFA) reduce the development of dexamethasone induced gastric damage.[4] Similarly, vitamin E present abundantly in fish oils is reported to reduce the development of indomethacin induced gastric damage.[5]

Cod liver oil is obtained from the liver of the cod fish and is different from fish oil. The main difference is that CLO is rich in vitamin A and vitamin D, as compared to fish oil and both these vitamins have therapeutic effects.[6,7] Earlier studies carried out with CLO indicate that CLO inhibits the development of indomethacin induced gastric ulcers in rats.[4] However, other studies suggest that CLO may inhibit the formation of arachidonic acid and may thus decrease the production of cytoprotective prostaglandins leading to the development of gastric ulcers, especially under conditions of stress.[8] The exact effect of CLO administration on the healing or development of gastric ulcer is not known. Since CLO is widely used as a dietary supplement, the present study was undertaken to evaluate its effect on the development and healing of gastric and duodenal ulcers in rats.

## Materials and Methods

### Experimental animals

Male albino Wistar rats weighing between 200 and 250 gm were used. The experimental protocol was approved by the Institutional Animal Ethics Committee. The animals were maintained under standard conditions in an animal house approved by the Committee for the Purpose of Control and Supervision on Experiments on Animals (CPCSEA).

### Drugs and chemicals

Acetic acid (E-Merck, Mumbai, India), cysteamine hydrochloride (Hi-media, Mumbai, India), ethanol (Bengal Chemicals, Kolkatta, India), indomethacin (Sigma, St. Louis, MO, USA), ketamine hydrochloride (Troika Parentrals, Gujarat, India) and CLO (Universal Medicare Pvt. Ltd., Mumbai, India).

### Determination of purity of CLO

The monographic analysis of CLO was carried out using procedures mentioned in the British Pharmacopoeia,[9] the United States Pharmacopoeia[10] and the Indian Pharmacopoeia.[11]

## Gastric Ulcers

### Acetic acid-induced chronic gastric ulcer

The method described by Okabe *et al.* (1970)[12] was followed. The animals were fasted for 24 h prior to the experiment. Under light ether anaesthesia, ulcers were induced by applying glacial acetic acid (0.05 ml) over the anterior serosal surface of the stomach for 60 seconds. The animals were treated with ranitidine (50 mg/kg, p.o.),[13] low dose of CLO (0.5 g/kg p.o.)[14] or high dose of CLO (1 g/kg p.o.),[14] once daily, for 10 days after the induction of ulcer, while the control group received only the vehicle. The rats were sacrificed on the 10^th^ day, the stomachs removed and cut open along the greater curvature.

The ulcer index was determined using the formula:[15]

Ulcer index=10/X

Where X = Total mucosal area/Total ulcerated area.

Based on their intensity, the ulcers were given scores as follows:

0 = no ulcer, 1 = superficial mucosal erosion, 2 = deep ulcer or transmural necrosis, 3 = perforated or penetrated ulcer.

### Pylorus ligation induced ulcers

The ligation of pyloric end of the stomach was performed on animals fasted for 36 h, under ether anaesthesia.[16,17] The two doses of CLO or ranitidine was administered intraduodenally, immediately after pylorus ligation. The animals were sacrificed six hours after pylorus ligation, through an overdose of ether anaesthesia. The stomachs were isolated and the content collected and centrifuged. The volume of the gastric juice was measured and this was used for the estimation of free acidity,[18] total acidity[18] pepsin content[19] and total proteins.[20] The ulcer index and gastric mucous content was determined.[21]

### Healing of indomethacin induced gastric ulcers

The gastric ulcers were induced by administering indomethacin (5 mg/kg. p.o.) for five days.[22] The animals were then treated either with misoprostol (100 *µ*g/kg, p.o),[23] low dose of CLO (0.5 g/kg p.o.) or high dose of CLO (1 g/kg p.o.), once daily for another five days, after the induction of ulcer, while the control group received only vehicle. The rats were sacrificed on the fifth day and the ulcer index was determined. The glandular portion of the stomach was taken and used for estimation of mucin content,[21] total proteins,[20] antioxidant factor's super oxide dismutase activity[24] and catalase activity.[25]

### Ethanol induced ulcers

All the animals were fasted for 36 h before the administration of ethanol. The standard drug (misoprostol 100 *µ*g/kg, p.o.) or CLO was administered one hour before ethanol administration. Ethanol (90%) was administered to all the animals at a dose of 1 ml/200gm and after one hour, all the animals were sacrificed and ulcer index was determined as mentioned above.[26]

### Cold restraint stress induced ulcers

The ulcer was induced by subjecting the animals to cold restraint stress. Ranitidine or CLO was administered 30 min prior to subjecting the animals to stress. The animals were placed in a restraint cage and the cage was placed at a temperature of 2°C for 3 h. The animals were sacrificed after three hours and the ulcer index was determined.[27,28]

## Duodenal Ulcers

### Cysteamine induced duodenal ulcers

Duodenal ulcer was induced by administering cysteamine hydrochloride (400 mg/kg, p.o.) twice, at an interval of four hours. Ranitidine or CLO was administered 30 min prior to each dose of cysteamine hydrochloride. After 24 h, all the animals were sacrificed and the duodena were excised carefully and cut open along the antimesentric side. The duodenal ulcer area, ulcer score and ulcer index were determined.[29]

Based on their intensity, the ulcers were given scores as follows:

0 = no ulcer, 1 = superficial mucosal erosion, 2 = deep ulcer or transmural necrosis,

3 = perforated or penetrated ulcer.

The ulcer index was calculated using the following equation:[30]

$$\text{Ulcer index}=\frac{\text{Arithmetic mean of intensity in a group}+\text{number of ulcer positive animals}}{\text{Total number of animals}}\times 2$$

### Statistical analysis

The statistical significance was assessed using one-way analysis of variance (ANOVA) followed by Bonferrroni's comparison test. For comparing nonparametric ulcer scores, anova followed by non-parametric Dunn post test was used. The values are expressed as mean ± SEM and *P* < 0.05 was considered significant.

## Results

### Determination of purity of CLO

The monographic analysis of CLO revealed the following results:

Saponification value - 185.13
Acid value - 1.12
Refractive index - 1.475

The assay of vitamin A was carried out as per the United States Pharmacopoeia and the vitamin A content was 2133 IU /ml.

The assay of vitamin D was carried out using the HPLC method mentioned in the Indian Pharmacopoeia and the CLO sample was found to contain 114 IU/ml of vitamin D.

All the values are within the limits prescribed by the pharmacopoeia.

### Acetic acid-induced chronic gastric ulcer

The CLO produced a significant reduction in the ulcer index and the ulcer score. Both the high dose (1 g/kg, p.o.) and the low dose of CLO (0.5 g/kg, p.o.) were effective in reducing the ulcer index and the ulcer score, when compared to control. The high dose of CLO was significantly more effective than the low dose in reducing the ulcer index. Ranitidine was most effective in reducing both ulcer score and ulcer index [Table 1].

**Table 1:** Effect on acetic acid induced chronic gastric ulcers

*Treatment*	*Ulcer score*	*Ulcer index*
Control	2.8 ± 0.2000	0.508 ± 0.0231
CLO (0.5 g/kg, p.o.)	1.8 ± 0.2000*	0.334 ± 0.0177*
CLO (1 g/kg, p.o.)	1.2 ± 0.2000*	0.214 ± 0.0050*+
Ranitidine (50 mg/kg, p.o.)	0.0 ± 0.000*	0.028 ± 0.0115*
F-value	26.83	158.37

CLO = Cod liver oil, All values are mean ± SEM, n = 5-6.

* *P*<0.05 vs control

+ *P*<0.05 vs low dose.

### Pylorus ligation induced gastric ulcers

Both doses of CLO produced a significant reduction in the ulcer index, free acidity, total acidity and pepsin content, when compared to control. The effects observed were dose dependent, with high dose of CLO (1 g/kg, p.o.) showing more effect, as compared to the low dose (0.5 g/kg, p.o.). The mucin content and total protein were significantly increased by both doses of CLO in a dose dependent manner, when compared to control. Ranitidine also showed similar effects, but was more effective compared to both doses of CLO [Table 2].

**Table 2:** Effect on free acidity, total acidity, ulcer index mucin content, pepsin content and total proteins in pylorus ligated rats

*Treatment*	*Free acidity (mEq/litre)*	*Total acidity (mEq/litre)*	*Ulcer index*	*Mucin content (*µ*g/g)*	*Pepsin content (*µ*mol/6hr)*	*Total protein (mg/ml)*
Control	47.20 ± 2.577	64.80 ± 4.598	0.908 ± 0.010	1.528 ± 0.219	0.774 ± 0.020	9.76 ± 0.654
CLO (0.5 g/kg, p.o.)	35.80 ± 2.010*	41.20 ± 2.746*	0.478 ± 0.020*	2.672 ± 0.234*	0.238 ± 0.009*	16.39 ± 0.913*
CLO (1 g/kg, p.o.)	24.40 ± 1.030*+	28.80 ± 0.969*+	0.236± 0.013*+	4.798 ± 0.023*+	0.184 ± 0.009*	23.58 ± 0.997*+
Ranitidine (50 mg/kg, p.o.)	16.60 ± 1.435*	22.40 ± 0.927*	0.064 ± 0.005*	4.242 ± 0.012*	0.108 ± 0.012*	30.40 ± 1.841*
F-value	56.88	53.64	818.94	104.53	528.7	49.98

CLO = Cod liver oil. All values are mean ± SEM, n = 5-6.

* *P*<0.05 vs control

+ *P*<0.05 Vs low dose.

### Healing of indomethacin induced gastric ulcers

The healing of indomethacin induced gastric ulcers was significantly increased by both doses of CLO, as indicated by a reduction in the ulcer index. Both the doses of CLO also produced a significant increase in mucin content, superoxide dismutase and catalase activity, with the high dose showing more activity, as compared to the low dose. The standard drug misoprostol reduced the ulcer index and increased gastric mucus secretion, but did not affect the protein content or enzyme activity [Table 3].

**Table 3:** Effect on mucin content, ulcer index, total proteins, anti oxidant factors in indomethacin induced ulcers

*Treatment*	*Mucin content *µ*g/g*	*Ulcer index*	*Total protein (mg/ml)*	*SOD Units of proteins*	*Catalase Units/mg of proteins*
Control	0.556 ± 0.111	0.730 ± 0.015	17.84 ± 0.936	41.30 ± 3.597	38.76 ± 3.452
CLO (0.5 g/kg, p.o.)	1.671 ± 0.193*	0.424 ± 0.011*	40.94 ± 3.577*	96.10 ± 6.254*	84.72 ± 3.176*
CLO (1 g/kg, p.o.)	2.686 ± 0.227*+	0.174 ± 0.008*+	57.90 ± 3.794*+	126.20 ± 5.687*+	109.70 ± 6.414*+
Misoprostol (100 µg/kg, p.o.)	2.303 ± 0.191*	0.080 ± 0.008*	26.04 ± 0.106	46.46 ± 4.816	43.16 ± 3.456
F-value	23.05	761.23	44.58	62.68	58.28

CLO = Cod liver oil. All values are mean ± SEM, n = 5-6.

* *P*<0.05 Vs control

+ *P*<0.05 Vs low dose.

### Ethanol induced and cold restraint stress induced ulcers

Both the doses of CLO showed a significant reduction in the ulcer index, when compared to control. Misoprostol and ranitidine showed more reduction in the ulcer index than CLO, in ethanol induced and cold restraint stress induced ulcer models respectively [Tables 4 and 5]

**Table 4:** Effect on ulcer index in ethanol induced gastric ulcers

*Treatment*	*Ulcer index*
Control	1.175 ± 0.1116
CLO (0.5 g/kg, p.o.)	0.275 ± 0.0154*
CLO (1 g/kg, p.o.)	0.178 ± 0.0175*
Misoprostol (100*µ*g/kg, p.o.)	0.161 ± 0.0127*
F-value	87.41

All values are mean ± SEM, n = 5-6

* *P*<0.05 vs control.

**Table 5:** Effect on ulcer index in cold restraint stress induced gastric ulcers

*Treatment*	*Ulcer index*
Control	0.732 ± 0.055
CLO (0.5 g/kg, p.o.)	0.210 ±0.013*
CLO (1 g/kg, p.o.)	0.098 ± 0.005*
Ranitidine(50 mg/kg, p.o.)	0.068 ± 0.003*
F-value	139.14

CLO = Cod liver oil, All values are mean ± SEM, n = 5-6,

* *P*<0.05 vs control

### Cysteamine induced duodenal ulcers

Both the doses of CLO produced significant reduction in the ulcer index, the ulcer area and the ulcer score, when compared to control. The high dose (*P*< 0.001) of CLO showed more significant effect in ulcer area and ulcer score than the low dose. Ranitidine completely prevented the development of duodenal ulcers. The ulcer was developed in the anti-mesenteric side of the duodenum [Table 6].

**Table 6:** Effect on ulcer area, ulcer score and ulcer index in cysteamine induced duodenal ulcers

*Treatment*	*Ulcer index*	*Ulcer area*	*Ulcer score*
Control	4.0	38.0 ± 1.449	2.0 ± 0.000
CLO (0.5g/kg, p.o.)	2.6	25.4 ±3.234*	1.2 ± 0.200*
CLO (1g/kg, p.o.)	1.2	14.6 ± 2.040*+	0.4 ± 0.244*+
Ranitidine			
(50mg/kg, p.o.)	0	0.0 ± 0.000*	0.0 ± 0.000*
F-value	68.36	29.63	

CLO = Cod liver oil, All values are mean ± SEM, n = 5-6,

* *P*<0.05 vs control,

+ *P*<0.05 vs low dose.

## Discussion

The present study investigated the effect of CLO on the gastric and duodenal ulcers. Cod liver oil showed effect on the healing of gastric ulcers induced by acetic acid and prevented the development of gastric ulcers induced by different methods and cysteamine induced duodenal ulcer.

Application of glacial acetic acid (0.05ml) on the serosal surface of the stomach produced deep penetrating gastric ulcer that resembles human peptic ulcer disease. Since the healing process of this ulcer closely resembles that of human peptic ulcers, this model is quite useful for studying the effect of drugs on the healing of peptic ulcers.[31] CLO was effective in augmenting the gastric ulcer healing in this model. To evaluate the mechanism by which CLO increased gastric ulcer healing, further studies were carried out using other models of gastric ulcers.

Pylorus ligation induced ulcer is one of the most widely used methods for studying the effect of drugs on gastric secretion. Agents that decrease gastric acid secretion and/or increase mucus secretion are effective in preventing the ulcers induced by this method. The ligation of the pyloric end of the stomach causes accumulation of gastric acid in the stomach, leading to the development of ulcers in the stomach. The original Shay rat model involves fasting of rats for 72 h, followed by ligation of the pyloric end of the stomach for 19 h.[16] In the present study, the modification of Shay rat model described by Kulkarni[17] was followed, which involves fasting of the animals for 36 h and pyloric ligation only for six hours. Ranitidine and CLO significantly decreased the secretion of gastric aggressive factors, free acidity, total acidity and pepsin content, and increased the mucus secretion, which is cytoprotective.

Ethanol induced gastric ulcer and indomethacin induced gastric ulcers were employed to study the cytoprotective effect of CLO. Ethanol induces gastric lesion, due to its corrosive effect. It rapidly penetrates the gastric mucosa, causing cell and plasma membrane damage, leading to increased membrane permeability to sodium and water. It also produces massive intracellular accumulation of calcium, which represents a major step in the pathogenesis of gastric mucosal injury. This leads to cell death and exfoliation in the surface epithelium.[32,33] Both the doses of CLO were effective in preventing development of ethanol induced gastric ulcers, indicating that CLO possesses gastric cytoprotective effect.

Indomethacin produces erosions and ulcers in the stomach due to the inhibition of prostaglandin synthesis.[34] The gastric cytoprotective agents are effective in preventing ulcers induced by indomethacin. Like ethanol induced gastric ulcers, CLO was effective in reducing ulcer index and in increasing the mucus content. Further, CLO produced an increased activity of antioxidant enzymes, suggesting that its effect on ulcer may partly be due to its antioxidant action.

The pathophysiology of stress-induced ulcers is complex; the ulcers are produced due to the release of histamine, leading to an increase in acid secretion and a reduction in mucus production.[35,36] Stress also causes an increase in gastrointestinal motility, which causes folds in the gastrointestinal tract The folds in the stomach are more susceptible to damage, when they come in contact with acid. The stress also brings the central nervous system into play. Agents that decrease G.I. motility, gastric secretion or those having central actions are helpful in reducing ulcers due to stress. Ranitidine and CLO were effective in reducing ulcers induced by stress. The reduction in stress induced gastric ulcers may probably be due to the reduction in gastric secretion, as there are no reports to indicate the effect of CLO on gastric motility or the central nervous system.

Cysteamine induced duodenal ulcer in rat resembles that of duodenal ulcer in humans, histopathologically and pathophysiologically. Cysteamine hydrochloride inhibits the alkaline mucus secretion from the Brunner's glands in the proximal duodenum and stimulates the rate of gastric acid secretion. Gastric emptying is also delayed and serum gastrin concentration is increased.[27]

Cod liver oil was effective in reducing the ulcer area and the ulcer score. The effect of fish oil on the development of gastric and duodenal ulcers has been studied extensively. Fish oil is reported to reduce gastric secretion and also prevent the development of indomethacin induced gastric ulcers.[37,38] Fish oil is also used as dietary supplements for ulcerative colitis.[39]

As mentioned earlier, CLO inhibits the development of indomethacin induced gastric ulcers in rats.[40] However, other studies suggest that CLO may inhibit the formation of arachidonic acid and may thus decrease the production of cytoprotective prostaglandnins, leading to the development of gastric ulcers, especially under conditions of stress.[3]

Although earlier reports on the effect of CLO on gastric ulcers are confusing, studies carried out using different constituents of fish oils such as omega-3-fatty acid and polyunsaturated fatty acids reveal that they possess antiulcer and antioxidant effect. Omega 3 triglycerides are also known to inhibit ulcer formation in pylorus ligated rats[41] and increase healing of gastric ulcers in rats.[42] Omega-3-fatty acid also possesses antioxidant effect.[43] Fish oil, rich in omega-3-fatty acid, is reported to inhibit offensive mucosal factors and oxidative stress and to augment defensive mucosal factors.[44] Further, fish oil reduced development of cold plus restraint ulcers in rats.[45]

Polyunsaturated fatty acids (PUFA) are reported to reduce the development of dexamethasone induced and indomethacin induced gastric ulcers healing.[4,46] Moreover, decrease in intake of polyunsaturated fatty acid was shown to be associated with increased incidence of duodenal ulcers in humans.[47]

The exact constituents and the mechanism by which CLO reduced gastric and duodenal ulcer formation and increased gastric ulcer cannot be explained by the present data. However, it is speculated that the polyunsaturated fatty acid and the omega-3-fatty acids present abundantly in the CLO may be responsible for this effect. Furthermore, the antioxidant effect of CLO may also be responsible for its anti-ulcer action.[48]

The results of the present investigation suggest that consumption of CLO is beneficial for patients suffering from peptic ulcer disease. Cod liver oil may produce both gastric antisecretory and gastric cytoprotective effect, resulting in increased healing of gastric and duodenal ulcers. However, the effect of CLO on the growth of *Helicobacter pylori*, one of the main causes for the development of gastric and duodenal ulcer, is not known. Studies on the effect of CLO on the *Helicobacter pylori* infection have to be carried out to further support the beneficial effect of CLO in peptic ulcer patients.

## Conclusion

Cod liver oil has an antiulcer effect. It increased healing of acetic acid induced chronic gastric ulcers and prevented the development of gastric ulcers induced by pylorus ligation, ethanol, stress or indomethacin. Cod liver oil was also effective in preventing the development of cysteamine induced duodenal ulcers.
